# The associations of systemic inflammation and insulin resistance-related indicators with psychopathology and BDNF in patients with chronic schizophrenia

**DOI:** 10.3389/fpsyt.2026.1802167

**Published:** 2026-04-13

**Authors:** Yinghan Tian, Yu Zhuang, Longlong Sun, Jinyue Xue, Pei Tang, Yun Zhang, Lewei Liu, Xianhu Yao, Wenzheng Li, Lei Xia, Huanzhong Liu

**Affiliations:** 1 Department of Psychiatry, The Fourth Affiliated Hospital of Anhui Medical University, Hefei, Anhui, China; 2 Anhui Psychiatric Center, Anhui Medical University, Hefei, Anhui, China; 3 Department of Psychiatry, School of Mental Health and Psychological Sciences, Anhui Medical University, Hefei, Anhui, China; 4 Department of Psychiatry, Fuyang Third People’s Hospital, Fuyang, Anhui, China; 5 Department of Psychiatry, Ma’anshan Fourth People’s Hospital, Ma’anshan, Anhui, China; 6 Department of Psychiatry, Hefei Fourth People’s Hospital, Hefei, Anhui, China; 7 Anhui Provincial Key Laboratory for Brain Bank Construction and Resource Utilization, Hefei, Anhui, China

**Keywords:** BDNF, chronic schizophrenia, CTI, inflammation, insulin resistance, psychopathology, TyG index

## Abstract

**Background:**

The triglyceride-glucose (TyG) index and C-reactive protein-triglyceride-glucose index (CTI) are innovative indicators for assessing insulin resistance (IR) and inflammation, yet research on them in patients with schizophrenia remains limited. This study aimed to explore TyG index and CTI levels and their associations with psychopathology and brain-derived neurotrophic factor (BDNF) in patients with chronic schizophrenia (CS).

**Methods:**

This cross-sectional study was conducted across one general hospital and two psychiatric hospitals in Anhui Province, China. Socio-demographic information and hematological parameters were collected from participants, and their psychiatric and depressive symptoms were assessed using the Positive and Negative Syndrome Scale (PANSS) and the Calgary Depression Scale for Schizophrenia (CDSS), respectively.

**Results:**

A total of 324 patients with CS and 150 healthy controls (HCs) were enrolled in the study. Compared with HCs, patients had higher TyG index and CTI levels (all *P* < 0.001). Binary logistic regression analyses revealed that among patients, a high TyG index level was significantly associated with higher BDNF levels and lower negative factor scores of the PANSS, while a high CTI level was significantly associated with higher depression-hopelessness factor scores of the CDSS and lower negative factor scores of the PANSS (all *P* < 0.05).

**Conclusion:**

Patients with CS had higher levels of TyG index and CTI, which were significantly associated with the severity of negative and depressive symptoms, as well as BDNF levels. It is suggested that the integration of the TyG index and CTI into clinical monitoring for patients with CS is necessary.

## 1 Introduction

Schizophrenia is a severe psychiatric disorder characterized by significant disability, high relapse rates, and marked social dysfunction, affecting approximately 24 million individuals worldwide. Notably, data from the Global Burden of Disease study indicate that the global crude prevalence, incidence, and overall disease burden of schizophrenia increased significantly between 1990 and 2019 (1). In addition, compared with the general population, patients with schizophrenia exhibit a markedly higher prevalence of metabolic and cardiovascular comorbidities, which profoundly impair quality of life and elevate the risk of premature mortality (2).

Insulin resistance (IR), defined as an impaired response of peripheral tissues to insulin action, constitutes a major risk factor for the development of type 2 diabetes mellitus and cardiovascular diseases (3). In recent years, a growing body of evidence has suggested a significant association between IR and various psychiatric disorders, including depression and schizophrenia. For instance, a meta-analysis of 70 studies revealed that patients with depression had significantly higher IR levels, which persisted despite treatment (4). Moreover, several Mendelian randomization studies have indicated that IR may be causally related to schizophrenia or share common pathophysiological mechanisms with it (5). In clinical practice, immune-inflammatory activation is commonly observed to coexist with both IR and schizophrenia. For example, a cohort study from the United Kingdom including 2,627 participants demonstrated that inflammation was associated with an increased risk of psychotic experiences related to IR in young populations (6). Perry et al. also reported that elevated levels of specific inflammatory biomarkers, such as C-reactive protein and interleukin-6, were implicated in the pathogenesis of both IR and schizophrenia (7). Therefore, during the long-term medical management of patients with chronic schizophrenia (CS), it is necessary to employ a simple, sensitive measurement tool to identify patients at high risk of inflammation and IR, enabling early intervention.

The triglyceride-glucose (TyG) index, calculated using triglyceride (TG) and fasting blood glucose (FBG) levels, has been proposed as a novel biomarker for assessing IR levels in both the general population and patients with physical diseases (8, 9). Compared with the homeostasis model assessment of insulin resistance (HOMA-IR), the TyG index demonstrates comparable sensitivity and specificity, yet is more cost-effective (10). Furthermore, Ruan et al. incorporated C-reactive protein (CRP) into the TyG framework and developed a new inflammation-related IR indicator known as the C-reactive protein-triglyceride-glucose index (CTI) (11). CRP, a liver-derived acute-phase protein, reflects the activation of the systemic inflammatory response when elevated (12). Longitudinal studies have demonstrated that the increased CRP levels associated with schizophrenia may lead to differential alterations in white matter volume within specific regions of the cerebrum and cerebellum (13). Additionally, previous studies have also suggested associations between the TyG index and psychiatric disorders. For instance, a cross-sectional study and a meta-analysis have reported that higher TyG index levels are associated with an increased risk of depression and suicide (14, 15). Another study conducted in patients with first-episode drug-naïve major depressive disorder identified a non-linear association between the TyG index and psychotic symptoms, specifically that a positive association is evident when TyG index values exceed a defined threshold (16). Nevertheless, to the best of our knowledge, only one small-sample cross-sectional study has revealed that patients with schizophrenia exhibit significantly higher TyG index levels than healthy controls (HCs), thereby suggesting a potentially elevated cardiovascular risk in this patient population (17).

Brain-derived neurotrophic factor (BDNF) is widely distributed throughout the central nervous system and serves as a crucial regulatory factor that promotes neurogenesis, neural development, and the formation of synaptic connectivity and plasticity (18). A considerable number of studies have shown that BDNF is involved in the pathophysiological mechanisms of various neuropsychiatric disorders, including schizophrenia, mood disorders, and Alzheimer’s disease (19, 20). In terms of treatment implications, a recent systematic review indicated that baseline BDNF levels could effectively predict subsequent treatment response in patients with schizophrenia (21). Interestingly, BDNF also plays a pivotal role in the maintenance of energy homeostasis and the regulation of metabolism. Early experimental studies suggested that BDNF knockout mice exhibited overeating and weight gain (22), whereas administration of BDNF reduced food intake, lowered FBG levels, and improved IR in diabetic and obese mice (23, 24). However, recent clinical studies have reported inconsistent findings. For example, a recent meta-analysis found that patients with type 2 diabetes exhibited significantly lower BDNF levels than HCs, with no significant association detected between BDNF levels and HOMA-IR (25). In patients with CS, Yang et al. reported the positive association between BDNF levels and HOMA-IR exclusively in females (26).

Currently, the clinical significance of the TyG index and CTI in schizophrenia remains elusive, with inconsistent findings reported across previous studies. Therefore, this study aimed to (1) examine the differences in TyG index and CTI levels between patients with CS and HCs, and (2) explore the associations of TyG index and CTI with psychopathology and BDNF levels.

## 2 Methods

### 2.1 Study design and participants

This multicentre cross-sectional study was conducted in central Anhui Province, China, involving one general hospital (the Fourth Affiliated Hospital of Anhui Medical University) and two psychiatric hospitals (the Fourth People’s Hospital of Hefei and the Fourth People’s Hospital of Ma’anshan). Patients were eligible for inclusion if they met the following criteria (1): aged between 18 and 75 years (2); diagnosed with schizophrenia by two senior psychiatrists via structured clinical interviews based on the Diagnostic and Statistical Manual of Mental Disorders, 5th edition (DSM-5) (3); duration of illness ≥ 5 years. The exclusion criteria included (1): any other psychiatric disorders (2); recent use of any non-steroidal anti-inflammatory drugs, corticosteroids, or other immunomodulatory agents (3); infectious or immune system diseases, or other serious physical diseases (4); pregnant or breastfeeding women. The HCs were all recruited from the medical examination center of the Fourth Affiliated Hospital of Anhui Medical University, were age- and sex-matched with the patient group, and had no personal or family history of psychiatric diseases.

The protocol of this study was approved by the Medical Ethics Committee of the Fourth Affiliated Hospital of Anhui Medical University (201805-kyxm-03) and was registered in the Chinese Clinical Trial Registry (ChiCTR1800017044). Participants and their guardians signed the written informed consent form after fully understanding the content and purpose of the study. All study procedures were conducted in accordance with the Declaration of Helsinki.

### 2.2 Data collection and measurements

#### 2.2.1 Socio-demographic characteristics

A predesigned questionnaire was used to collect socio-demographic and clinical data, including age (years), sex (male/female), education (years), smoking (yes/no), history of physical illness (yes/no), marital status (single/married), age of onset (years) and duration of illness (years). Body mass index (BMI) was calculated as weight (kg)/height (m)^2^. Due to the significant metabolic syndrome (MetS) effects of clozapine/olanzapine during treatment, patients on these two drugs were grouped separately, while the remaining patients were classified into the low MetS risk antipsychotics group (27, 28). Based on the Defined Daily Dose (DDD) conversion method recommended by the World Health Organization, the antipsychotic dose per patient was converted to chlorpromazine equivalents (29).

#### 2.2.2 Clinical measurements

Psychiatric symptoms of the patients included in this study were assessed using the Chinese version of the 30-item Positive and Negative Syndrome Scale (PANSS) (30). Each item was evaluated on a seven-point scale, ranging from 1 (none) to 7 (extremely severe), with a higher total score indicating more severe symptoms. The scale consists of five factors: positive factor (items P1, P3, P5, and G9), negative factor (items N1, N2, N3, N4, N6, and G7), cognitive factor (items P2, N5, and G11), excited factor (items P4, P7, G8, and G14), and depressive factor (items G2, G3, and G6) (31). The Calgary Depression Scale for Schizophrenia was used to assess depression in patients over the last two weeks, and it has good inter-rater reliability and internal consistency (32). The scale consists of 9 items that are scored on a 4-point scale ranging from 0 (absent) to 3 (severe), with higher total scores indicating more severe depressive symptoms. The two-factor model was used for the statistical scoring (33). The depression-hopelessness factor scores included items 1, 2, 6, 8, and 9. The self depreciation-guilt factor scores included items 3, 4, and 5.

#### 2.2.3 Hematological index measurements

Blood samples were collected from patients between 07:00 and 08:00 AM following an overnight fast. The blood samples were centrifuged at 3,600 rpm for 10 minutes, and separated to isolate serum samples. Then, the serum was immediately stored at -80 °C and tested within 30 days. FBG, TG, and CRP levels were measured by the oxidase method (Meikang Biotechnology Company, Zhejiang, China), the GPO-PAP method (Beijing Leadman Biochemical Company, Beijing, China), and the immunoturbidimetry method (Beijing Leadman Biochemical Company, Beijing, China). The TyG index was calculated using the formula: ln [TG (mg/dL) × FBG (mg/dL)/2]. The CTI was calculated using the formula: 0.412 × ln [CRP (mg/L)] + TyG. The enzyme-linked immunosorbent assay (ELISA) (Cusabio Biotechnology Company, Wuhan, China) was used to determine the level of BDNF, and the results were expressed as ng/mL. The BDNF levels were ln-transformed as the Kolmogorov-Smirnov test showed that they did not follow a normal distribution.

### 2.3 Statistical analysis

The continuous variables were described as mean ± standard deviation (SD) and the categorical variables were described as frequency distributions and percentages (%). The normal distribution of continuous variables was assessed using the Kolmogorov-Smirnov test. Patients were divided into low and high groups based on the 50th percentile of TyG index and CTI, respectively. Independent samples t-tests, Mann-Whitney U tests, and Chi-square tests were used to compare socio-demographic and clinical characteristics (e.g., type of antipsychotics and chlorpromazine equivalents) between the patients and HCs, between the low and high TyG index groups, and between the low and high CTI groups, respectively. Binary logistic regression models (Forward: LR) were used to examine the independent factors associated with TyG index and CTI in the patients, treating TyG index and CTI as separate dependent variables, and including variables significant in the univariate analyses (*P* < 0.05) as independent variables. Pearson or Spearman correlation analyses were used to examine the correlations of TyG index and CTI with other clinical data in the patients, respectively. Then multivariate linear regression models (Forward: LR) were used to examine any significant correlations (*P* < 0.05) identified in correlation analyses. Statistical Product and Service Solutions (SPSS) version 23.0 was used for statistical analyses. A two-tailed *P*-value < 0.05 was considered statistically significant.

## 3 Results

### 3.1 Comparisons between patients and HCs

**Figure 1:**
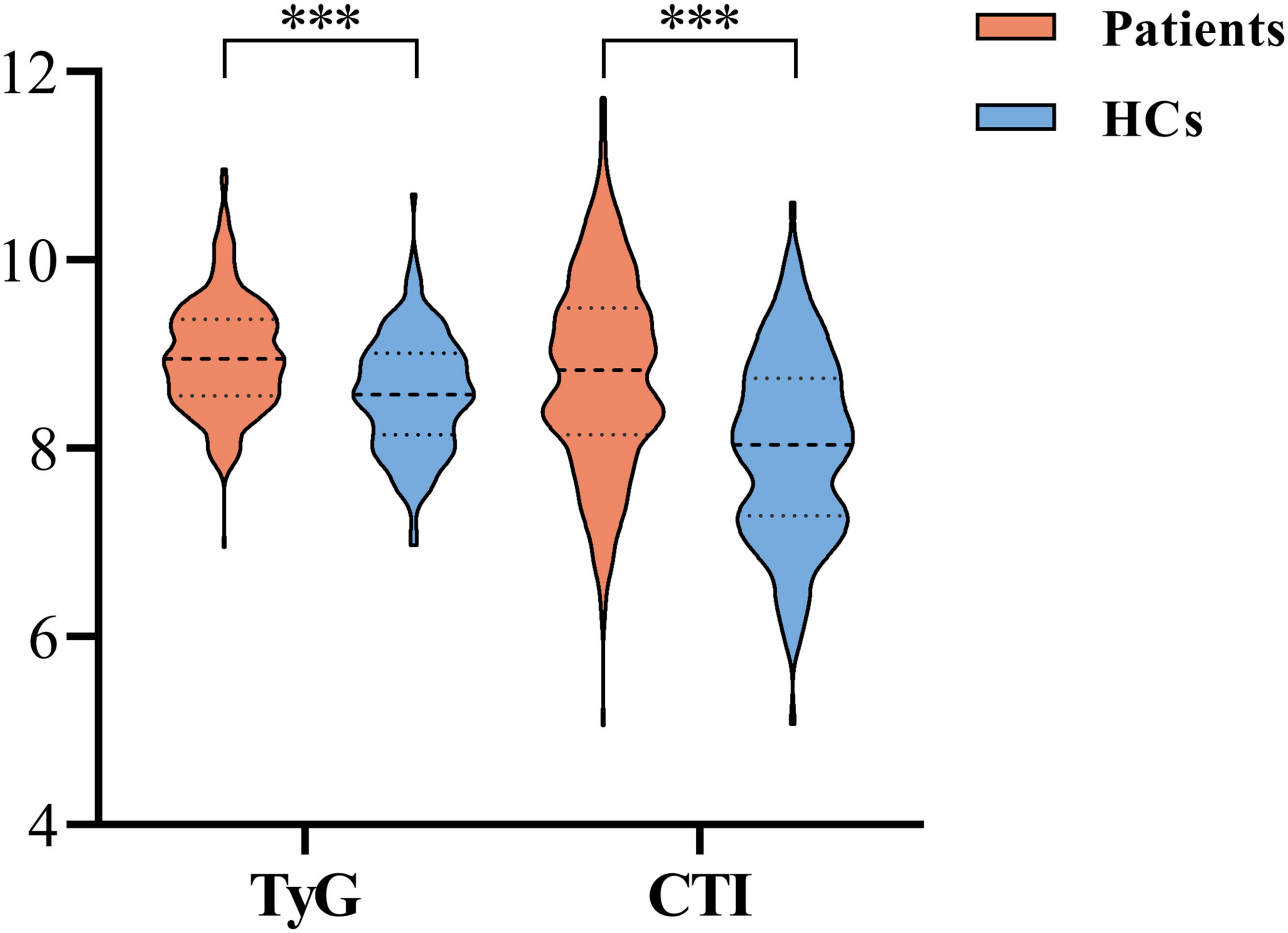
Comparison of TyG index and CTI between patients and HCs. (HCs, Healthy controls; TyG, triglyceride-glucose; CTI, C-reactive protein-triglyceride-glucose index. ^***^P<0.001).

A total of 324 patients with CS and 150 HCs completed the assessment and were included in the statistical analysis. As shown in **Table 1**, the mean age of the patients was 45.10 years (SD = 11.67), and 192 (59.3%) were male. Of these patients, 207 (63.9%) were receiving clozapine/olanzapine treatment. The patients had a lower education level, and higher BMI, TG level, CRP level, TyG index, and CTI than HCs (all *P* < 0.05). Analysis of covariance (ANCOVA) showed that differences in TyG index (*F* = 29.034, *P* < 0.001) and CTI (*F* = 38.486, *P* < 0.001) between the two groups remained significant after controlling for BMI and education (**Figure 1**).

**Table 1:** Socio-demographic and clinical characteristics of patients and healthy controls.

Variables	Patients (*N* = 324)	Healthy controls (*N* = 150)	Statistics	
	*N* (%)	*N* (%)	*χ^2^*	*P*
Male	192 (59.3)	79 (52.7)	1.820	0.177
Smoking	125 (38.6)	47 (31.3)	2.329	0.127
History of physical illness	62 (19.1)	–		
Marital status				
Single	93 (28.7)	–		
Married	231 (71.3)	–		
Type of antipsychotics				
Clozapine/olanzapine	207 (63.9)	–		
Low MetS risk antipsychotics	117 (36.1)	–		
	Mean (SD)	Mean (SD)	*t/Z*	*P*
Age (years)	45.10 ± 11.67	45.98 ± 13.32	0.731	0.465
BMI (kg/m^2^)	24.10 ± 3.84	22.53 ± 2.40	-5.359	**<0.001**
Education (years)	8.07 ± 3.62	9.68 ± 3.86	4.233	**<0.001**
Age of onset (years)	26.12 ± 8.30	–		
Duration of illness (years)	18.99 ± 10.47	–		
Chlorpromazine equivalents (mg/d)	455.81 ± 258.37	–		
TG (mg/dL)	198.11 ± 134. 02	135.87 ± 86.40	-6.681^a^	**<0.001**
FBG (mg/dL)	96.39 ± 24.68	93.37 ± 15.94	-1.598	0.111
CRP (mg/L)	2.45 ± 6.74	0.95 ± 2.37	-5.330^a^	**<0.001**
TyG	8.99 ± 0.59	8.58 ± 0.61	-6.907	**<0.001**
CTI	8.79 ± 1.03	8.01 ± 0.99	-7.815	**<0.001**
Ln BDNF (ng/mL)	0.25 ± 0.90	–		
CDSS total score	3.34 ± 3.40	–		
Depression-hopelessness factor score	1.89 ± 2.20	–		
Self depreciation-guilt factor score	1.17 ± 1.41	–		
PANSS total score	77.96 ± 24.34	–		
Positive factor score	10.63 ± 5.01	–		
Negative factor score	17.65 ± 7.05	–		
Cognitive factor score	9.04 ± 2.96	–		
Excited factor score	7.89 ± 3.67	–		
Depressive factor score	6.84 ± 2.78	–		

MetS, metabolic syndrome; BMI, body mass index; TG, triglyceride; FBG, fasting blood glucose; CRP, C-reactive protein; TyG, triglyceride-glucose; CTI, C-reactive protein-triglyceride-glucose index; BDNF, brain-derived neurotrophic factor; CDSS, Calgary Depression Scale for Schizophrenia; PANSS, Positive and Negative Syndrome scale; SD, standard deviation; a, Mann-Whitney U test; Bolded *P* values < 0.05. .

### 3.2 Comparison of low vs. high TyG index and low vs. high CTI groups

As shown in **Table 2**, the patients in the high TyG index group had a higher proportion of females, a higher prevalence of history of physical illness, a higher BMI, a higher BDNF level, and a lower score on the negative factor than those in the low TyG index group (all *P* < 0.05). The patients in the high CTI group had a higher proportion of using clozapine/olanzapine, a higher BMI, a higher BDNF level, a higher score on the depression-hopelessness factor, and a lower score on the negative factor than those in the low CTI group (all *P* < 0.05).

**Table 2:** Socio-demographic and clinical characteristics of patients in low and high TyG index and CTI groups.

Variables	TyG (categorical)				CTI (categorical)			
	Low group (6.95-8.95)	High group (8.96-10.97)	Statistics		Low group (5.06-8.82)	High group (8.84-11.73)	Statistics	
	*N* (%)	*N* (%)	*χ^2^*	*P*	*N* (%)	*N* (%)	*χ^2^*	*P*
Male	106 (65.4)	86 (53.1)	5.114	**0.024**	101 (62.3)	91 (56.2)	1.278	0.258
Smoking	64 (39.5)	61 (37.7)	0.117	0.732	59 (36.4)	66 (40.7)	0.638	0.424
History of physical illness	19 (11.7)	43 (26.5)	11.489	**0.001**	27 (16.7)	35 (21.6)	1.277	0.259
Marital status			2.549	0.110			0.739	0.390
Single	40 (24.7)	53 (32.7)			50 (30.9)	43 (26.5)		
Married	122 (75.3)	109 (67.3)			112 (69.1)	119 (73.5)		
Type of antipsychotics			3.010	0.083			11.251	**0.001**
Clozapine/olanzapine	96 (59.3)	111 (68.5)			89 (54.9)	118 (72.8)		
Low MetS risk antipsychotics	66 (40.7)	51 (31.5)			73 (45.1)	44 (27.2)		
	Mean (SD)	Mean (SD)	*t/Z*	*P*	Mean (SD)	Mean (SD)	*t/Z*	*P*
Age (years)	44.08 ± 12.24	46.12 ± 11.00	-1.575	0.116	44.37 ± 11.17	45.83 ± 12.13	-1.124	0.262
BMI (kg/m^2^)	22.73 ± 3.39	25.48 ± 3.78	-6.915	**<0.001**	23.00 ± 3.46	25.20 ± 3.90	-5.374	**<0.001**
Education (years)	7.77 ± 3.74	8.38 ± 3.49	-1.536	0.125	7.77 ± 3.81	8.38 ± 3.42	-1.505	0.133
Age of onset (years)	25.64 ± 8.72	26.60 ± 7.85	-1.045	0.297	25.73 ± 8.16	26.50 ± 8.44	-0.830	0.407
Duration of illness (years)	18.62 ± 10.66	19.36 ± 10.31	-0.740^a^	0.447	18.74 ± 9.64	19.24 ± 11.27	-0.429	0.668
Chlorpromazine equivalents (mg/d)	474.00 ± 266.12	437.62 ± 249.88	-1.373^a^	0.170	474.04 ± 268.94	437.59 ± 246.84	-1.223^a^	0.221
Ln BDNF (ng/mL)	0.07 ± 0.80	0.44 ± 0.96	-3.735	**<0.001**	0.11 ± 0.84	0.40 ± 0.94	-2.960	**0.003**
CDSS total score	3.27 ± 3.49	3.40 ± 3.31	-0.706^a^	0.480	2.92 ± 2.95	3.75 ± 3.76	-1.806^a^	0.071
Depression-hopelessness factor score	1.83 ± 2.26	1.94 ± 2.15	-0.917^a^	0.359	1.59 ± 1.87	2.19 ± 2.45	-1.972^a^	**0.049**
Self depreciation-guilt factor score	1.17 ± 1.38	1.17 ± 1.45	-0.136^a^	0.892	1.06 ± 1.29	1.28 ± 1.53	-1.220^a^	0.223
PANSS total score	79.93 ± 22.73	75.99 ± 25.76	1.457	0.146	78.95 ± 23.77	76.97 ± 24.92	0.732	0.465
Positive factor score	10.79 ± 4.98	10.48 ± 5.05	0.565	0.573	10.32 ± 4.87	10.94 ± 5.14	-1.120	0.263
Negative factor score	18.77 ± 7.06	16.52 ± 6.88	2.909	**0.004**	18.64 ± 7.00	16.65 ± 6.98	2.567	**0.011**
Cognitive factor score	9.16 ± 2.75	8.91 ± 3.17	0.749	0.454	9.24 ± 2.78	8.83 ± 3.13	1.238	0.217
Excited factor score	7.87 ± 3.57	7.91 ± 3.77	-0.106	0.916	7.84 ± 3.70	7.94 ± 3.65	-0.257	0.797
Depressive factor score	6.73 ± 2.69	6.96 ± 2.88	-0.738	0.461	6.80 ± 2.70	6.88 ± 2.88	-0.259	0.796

TyG, triglyceride-glucose; CTI, C-reactive protein-triglyceride-glucose index; MetS, metabolic syndrome; BMI, body mass index; BDNF, brain-derived neurotrophic factor; CDSS, Calgary Depression Scale for Schizophrenia; PANSS, Positive and Negative Syndrome scale; SD, standard deviation; a, Mann-Whitney U test; Bolded *P* values < 0.05. .

### 3.3 Factors associated with high TyG index and CTI (categorical)

As shown in **Table 3**, binary logistic regression analyses revealed that the high TyG index was significantly associated with a history of physical illness [odds ratio (OR) = 2.433, 95% confidence interval (CI): 1.275-4.643], higher BMI (OR = 1.194, 95% CI = 1.113-1.280) and BDNF level (OR = 1.466, 95% CI = 1.105-1.945), and a lower score on the negative factor (OR = 0.962, 95% CI = 0.929-0.997) (all *P* < 0.05). The analyses also showed that the high CTI was significantly associated with clozapine/olanzapine use (OR = 2.239, 95% CI = 1.366-3.671), higher BMI (OR = 1.166, 95% CI = 1.091-1.246) and score on the depression-hopelessness factor (OR = 1.198, 95% CI = 1.066-1.347), and a lower score on the negative factor (OR = 0.955, 95% CI = 0.922-0.990) (all *P* < 0.05).

**Table 3:** Independent factors associated with TyG index and CTI in patients.

Variables	*P*	OR	95% CI	
			Lower	Upper
TyG index (categorical)				
History of physical illness				
No	–	Reference	–	–
Yes	**0.007**	2.433	1.275	4.643
BMI (kg/m^2^)	**<0.001**	1.194	1.113	1.280
Ln BDNF (ng/mL)	**0.008**	1.466	1.105	1.945
Negative factor score	**0.033**	0.962	0.929	0.997
CTI (categorical)				
Type of antipsychotics				
Low MetS risk antipsychotics	–	Reference	–	–
Clozapine/olanzapine	**0.001**	2.239	1.366	3.671
BMI (kg/m^2^)	**<0.001**	1.166	1.091	1.246
Depression-hopelessness factor score	**0.003**	1.198	1.066	1.347
Negative factor score	**0.009**	0.955	0.922	0.990

TyG, triglyceride-glucose; CTI, C-reactive protein-triglyceride-glucose index; BMI, body mass index; BDNF, brain-derived neurotrophic factor; MetS, metabolic syndrome; OR, odds ratio; CI, confidence interval; Bolded *P* values < 0.05.

### 3.4 Independent correlates of TyG index and CTI (continuous)

Several socio-demographic and clinical variables were correlated with the TyG index and CTI in patients (**Supplementary Table 1**). Furthermore, multivariate linear regression analyses revealed that the TyG index was positively associated with BMI (*β* = 0.323, *P* < 0.001) and a history of physical illness (*β* = 0.176, *P* = 0.001), and negatively associated with score on the negative factor (*β* = -0.105, *P* = 0.042). The CTI was positively associated with age (*β* = 0.157, *P* = 0.002), BMI (*β* = 0.331, *P* < 0.001), clozapine/olanzapine use (*β* = 0.109, *P* = 0.032), and score on the depression-hopelessness factor (*β* = 0.146, *P* = 0.005), and negatively associated with score on the negative factor (*β* = -0.135, *P* = 0.012) (**Supplementary Table 2**). Notably, these associations observed using continuous variables were largely consistent with the group differences identified in the dichotomized analysis, reinforcing the robustness of the findings.

## 4 Discussion

To the best of our knowledge, this is the first study to investigate the TyG index and CTI levels in Chinese patients with CS, and their associations with psychopathology and BDNF levels. We found that the TyG index and CTI levels were significantly higher in patients with CS than in HCs. Similarly, two recent meta-analyses involving patients with schizophrenia reported that, compared with HCs, the patient group exhibited higher levels of IR and inflammatory proteins (34, 35). Previous studies have attempted to explain the potential associations between these phenomena from a variety of perspectives. First, regarding lifestyle factors, sedentary behavior and unhealthy dietary patterns are widely recognized as traditional risk factors associated with metabolic disturbances in patients with schizophrenia (36). Second, several studies on the gut microbiota have also provided novel insights. For example, a cross-sectional study from Poland indicated that patients with CS had altered gut microbiota composition, which was associated with IR, elevated inflammatory markers, and more severe negative symptoms (37).

Consistent with previous studies in the general population, our results revealed positive associations of the TyG index with BMI and physical comorbidities in patients with CS (38, 39). One possible explanation is that IR reduces the activity of hormone-sensitive lipase, and this reduction is potentially linked to increased fat storage and subsequent weight gain (40). Furthermore, a large-scale longitudinal study based on Bayesian network analysis indicated that elevated TyG index levels primarily increase cardiovascular disease risk, with this effect mainly mediated by overweight/obesity (41). In addition, we found that CTI was positively associated with age and clozapine/olanzapine use among patients with CS. It is well established that inflammatory processes escalate with increasing age (42). Specifically, the aging process affects macrophage function, thereby enhancing the production of pro-inflammatory cytokines, which in turn stimulate hepatic secretion of CRP (43). However, current evidence regarding the association between antipsychotics and inflammation remains conflicting. An early meta-analysis indicated that patients with schizophrenia showed no significant changes in CRP levels, irrespective of treatment with typical or atypical antipsychotics (44). In contrast, our previous study demonstrated that, across both animals and humans, clozapine administration was associated with higher inflammation levels and more severe metabolic disturbances (45). Similarly, a 12-month follow-up study found that patients with schizophrenia receiving olanzapine treatment exhibited a significant increase in CRP levels (46). Future large-scale longitudinal studies are warranted to elucidate the complex associations of specific antipsychotics with metabolic disturbances and inflammation.

In terms of psychopathology, this study revealed that the TyG index and CTI were negatively correlated with the negative factor scores. Similarly, a recent study reported that patients with schizophrenia in remission exhibited significantly higher TyG index levels and a higher proportion of clozapine/olanzapine use than those presenting with negative symptoms (17). This suggests that the negative associations between these indices and the negative factor scores may be attributable to antipsychotic treatment. For instance, a recent meta-analysis reported that clozapine and olanzapine significantly improve clinical outcomes in patients with schizophrenia compared to other antipsychotics (47). Nevertheless, these two agents are concomitantly associated with more pronounced metabolic side effects. Additionally, Kim et al. indicated that elevated lipid levels may contribute to a better antipsychotic response by altering the pharmacokinetics of certain antipsychotics, particularly clozapine (48). Furthermore, neuroimaging studies have suggested that negative symptoms in schizophrenia are associated with dysfunction in the reward system (49). Therefore, we hypothesize that patients with less severe negative symptoms may have relatively preserved reward system function, rendering them more prone to perceiving food as a rewarding stimulus and thus leading to excessive food intake and IR (50). Interestingly, consistent with findings from a national survey of general adults, our study also found that the CTI level was positively associated with the depression-hopelessness factor score (51). As a composite indicator, the CTI not only reflects IR status but also represents the body’s overall inflammatory level. A cross-sectional investigation indicated that elevated CRP levels are predictive of psychological distress (e.g., depression and anxiety) among patients with schizophrenia (52). In addition, prospective clinical trials and animal studies have demonstrated that anti-inflammatory treatments could alleviate depressive symptoms in patients and depression-like behaviors in mice (53, 54). Sălcudean et al. proposed that inflammatory responses may contribute to the development and persistence of depressive symptoms by disrupting neurotransmitter systems, activating the hypothalamic-pituitary-adrenal axis, and altering gut microbiota composition (55).

Finally, our study also identified a significant positive association between the TyG index and BDNF levels, which aligns with results from a Thai study of the child population (56). Miksza et al. further explored the relationship between glucose homeostasis parameters and BDNF concentrations, stratified by BDNF single nucleotide polymorphisms (57). The results showed that HOMA-IR was positively associated with BDNF levels in individuals with the GG genotype, whereas no such relationship was observed in those with the AA or AG genotypes. Conversely, an animal study demonstrated that IR model rats had decreased BDNF levels in the prefrontal cortex and striatum (58). Another two systematic reviews have indicated that individuals with diabetes or metabolic syndrome typically display reduced BDNF levels (59, 60). Elevated BDNF concentrations have been suggested to improve insulin sensitivity by reducing visceral fat accumulation and enhancing pancreatic β-cell function (57). Therefore, we hypothesize that the positive association between the TyG index and BDNF levels may reflect a compensatory mechanism, wherein BDNF levels increase adaptively alongside early IR in patients with CS, potentially helping to counteract metabolic impairment and maintain glucose homeostasis. Given the complex interplay between metabolic processes and BDNF regulation, future studies are needed to elaborate on these associations across diverse genotypes, disease subtypes, and populations.

This study has several limitations that should be acknowledged. First, due to the cross-sectional design, the observed associations of the TyG index, CTI, psychopathology, and BDNF levels should be interpreted as correlational rather than causal. Second, this study included only inpatients with CS, with no inclusion of patients with schizophrenia in other disease stages or those residing long-term in the community. Future studies could employ larger sample sizes to examine the differences in TyG index and CTI levels across different subgroups, along with their contributing factors. Third, the lack of systematic assessment of lifestyle factors (e.g., diet, physical activity, and sedentary behavior) in relation to the TyG index and CTI limits a more comprehensive interpretation of the results. Finally, the patients with CS had been receiving long-term antipsychotic treatment. Although we controlled for medication type and dosage in the analysis, potential confounding effects due to cumulative drug exposure and interindividual differences in drug metabolism could not be entirely ruled out.

## 5 Conclusion

In summary, compared with HCs, patients with CS had significantly higher TyG index and CTI levels. Patients with high TyG index and CTI exhibited lower severity of negative symptoms. The study also revealed independent positive associations between the TyG index and BDNF levels, as well as between CTI and the depression-hopelessness factor score. These findings carry important implications for explaining the potential role of inflammation and IR in schizophrenia. Given our results are preliminary, further research is warranted to investigate whether interventions targeting the TyG index and CTI could improve clinical outcomes in this population.

## Data Availability

The original contributions presented in the study are included in the article/**Supplementary Material**. Further inquiries can be directed to the corresponding authors.
