# Supplementary material for: The associations of systemic inflammation and insulin resistance-related indicators with psychopathology and BDNF in patients with chronic schizophrenia
Source: Front Psychiatry. 2026 Apr 13;17:1802167. doi: 10.3389/fpsyt.2026.1802167 (PMC13111298; doi:10.3389/fpsyt.2026.1802167)
Supplement: Supplementary file 1 [file SupplementaryFile1.docx]

**Supplementary Appendix**

**Table of Contents**

**Supplementary Table 1** Correlations of socio-demographic and clinical variables with TyG index and CTI in patients.

**Supplementary Table 2** Independent correlates of TyG index and CTI in patients.

**Supplementary TABLE 1** Correlations of socio-demographic and clinical variables with TyG index and CTI in patients.

| **Variables** | **TyG index (continuous)** | | **CTI (continuous)** | |
| --- | --- | --- | --- | --- |
|  | ***r*** | P | ***r*** | P |
| Age (years) | 0.065 | 0.244 | 0.138 | **0.013** |
| Sex (male/female) | -0.099 | 0.074 | -0.082 | 0.142 |
| BMI (kg/m^2^) | 0.366 | **<0.001** | 0.358 | **<0.001** |
| Education (years) | 0.118 | **0.034** | 0.052 | 0.348 |
| Smoking (yes/no) | 0.003 | 0.963 | 0.010 | 0.863 |
| History of physical illness (yes/no) | 0.226 | **<0.001** | 0.167 | **0.003** |
| Marital status (single/married) | 0.047 | 0.401 | 0.010 | 0.859 |
| Age of onset (years) | 0.025 | 0.659 | 0.107 | 0.054 |
| Duration of illness (years) | 0.045^a^ | 0.417 | 0.065^a^ | 0.245 |
| Type of antipsychotics (low MetS risk antipsychotics / clozapine/olanzapine) | 0.022 | 0.689 | 0.128 | **0.021** |
| Chlorpromazine equivalents (mg/d) | -0.102^a^ | 0.067 | -0.107^a^ | 0.054 |
| Ln BDNF (ng/mL) | 0.155 | **0.005** | 0.150 | **0.007** |
| CDSS total score | 0.040^a^ | 0.471 | 0.122^a^ | **0.029** |
| Depression-hopelessness factor score | 0.057^a^ | 0.304 | 0.133^a^ | **0.017** |
| Self depreciation-guilt factor score | -0.018^a^ | 0.741 | 0.077^a^ | 0.167 |
| PANSS total score | -0.106 | 0.057 | -0.083 | 0.135 |
| Positive factor score | -0.050 | 0.368 | -0.014 | 0.796 |
| Negative factor score | -0.163 | **0.003** | -0.142 | **0.011** |
| Cognitive factor score | -0.102 | 0.068 | -0.098 | 0.079 |
| Excited factor score | -0.013 | 0.810 | -0.032 | 0.564 |
| Depressive factor score | 0.019 | 0.729 | 0.018 | 0.745 |
| TyG, triglyceride-glucose; CTI, C-reactive protein-triglyceride-glucose index; BMI, body mass index; MetS, metabolic syndrome; BDNF, brain-derived neurotrophic factor; CDSS, Calgary Depression Scale for Schizophrenia; PANSS, Positive and Negative Syndrome scale; a, Spearman correlation analysis; Bolded *P* values < 0.05. | | | | |

**Supplementary TABLE 2** Independent correlates of TyG index and CTI in patients.

| **Variables** | **B** | **SE** | ***β*** | ***P*** |
| --- | --- | --- | --- | --- |
| **TyG index (continuous)** | | | | |
| BMI (kg/m^2^) | 0.050 | 0.008 | 0.323 | **<0.001** |
| History of physical illness | 0.264 | 0.077 | 0.176 | **0.001** |
| Negative factor score | -0.009 | 0.004 | -0.105 | **0.042** |
| **CTI (continuous)** | | | | |
| Age (years) | 0.014 | 0.004 | 0.157 | **0.002** |
| BMI (kg/m^2^) | 0.089 | 0.014 | 0.331 | **<0.001** |
| Clozapine/olanzapine | 0.232 | 0.108 | 0.109 | **0.032** |
| Depression-hopelessness factor score | 0.068 | 0.024 | 0.146 | **0.005** |
| Negative factor score | -0.020 | 0.008 | -0.135 | **0.012** |
| TyG, triglyceride-glucose; CTI, C-reactive protein-triglyceride-glucose index; BMI, body mass index; SE, standard error. Bolded *P* values < 0.05. | | | | |
